# *Nitraria sibirica* Pall.: A Halophytic Resource for Antioxidant-Rich Functional Foods and Ecological Resilience

**DOI:** 10.3390/foods14091646

**Published:** 2025-05-07

**Authors:** Keyi Lu, Xinmei Zhang, Liping Zhao, Jikun Xu, Jianmei Li

**Affiliations:** 1 School of Food Science and Pharmaceutical Engineering, Nanjing Normal University, Nanjing 210023, China; 2 College of Biological and Pharmaceutical Engineering, Shandong University of Aeronautics, Binzhou 256600, China

**Keywords:** *Nitraria sibirica* Pall., halophyte, phytochemical diversity, anthocyanins, antioxidant activity, ACE inhibition

## Abstract

*Nitraria sibirica* Pall., a halophytic shrub native to arid and saline–alkaline ecosystems, represents a dual-purpose resource for ecological stabilization and functional food development. This review synthesizes current knowledge of its bioactive compounds and pharmacological properties, while identifying research gaps in stress-induced metabolic regulation. The plant contains diverse phytochemicals including phenolic glycosides (e.g., clovin), alkaloids (e.g., nitraramine), immunomodulatory polysaccharides, and anthocyanins, which collectively demonstrate superior antioxidant capacity (freeze-dried polysaccharides outperform Butylated Hydroxytoluene (BHT)), significant antihypertensive effects via angiotensin-converting enzyme (ACE) inhibition and nitric oxide (NO) pathway activation, and broad-spectrum antimicrobial activity against foodborne pathogens. Notably, its pectin components reduce allergen absorption by 72%, suggesting potential as hypoallergenic food additives. These findings validate traditional medicinal uses while revealing novel applications in functional foods and nutraceuticals. Despite promising preclinical results, key challenges remain in understanding compound synergies under environmental stress and translating findings to human applications. Future research should employ multi-omics approaches to elucidate stress-adaptive phytochemical biosynthesis, coupled with clinical validation and sustainable cultivation methods. As a model species for ecological and nutritional applications, *N. sibirica* offers innovative solutions for addressing both global health challenges (metabolic disorders) and environmental concerns (soil rehabilitation), positioning it at the forefront of climate-resilient agricultural innovation.

## 1. Introduction

The genus *Nitraria* (Zygophyllaceae) represents a remarkable group of halophytic shrubs that have evolved exceptional adaptations to extreme environments. Distributed across arid regions and high-altitude ecosystems of Eurasia, key species including *N. sibirica* Pall., *N. retusa*, *N. tangutorum* Bor., *N. roborowskii* Kom., and *Nitraria iliensis* sp. nov. thrive in challenging habitats ranging from saline–alkali soils to desertified sands and plateau margins [1,2,3]. Modern multi-omics approaches have elucidated sophisticated biochemical mechanisms underlying their resilience to multiple abiotic stressors, particularly ultraviolet radiation, hypoxia, and temperature extremes [4,5,6]. Phytochemical characterization of this genus has identified diverse bioactive metabolites, including flavonoids, alkaloids, and polysaccharides, many of which exhibit potent antifatigue, antitumor, anti-oxidative, and antimutagenic activities [7,8]. These specialized metabolites are associated with upregulated expression of core phenylpropanoid pathway genes (C4H, F3H, 4CL, DFR), which collectively enhance both reactive oxygen species (ROS) scavenging efficiency and osmoregulatory capacity. Such comprehensive adaptive responses establish *Nitraria* species as exemplary models for investigating plant–environment coevolution in extreme ecosystems [4,9].

**Figure 1:**
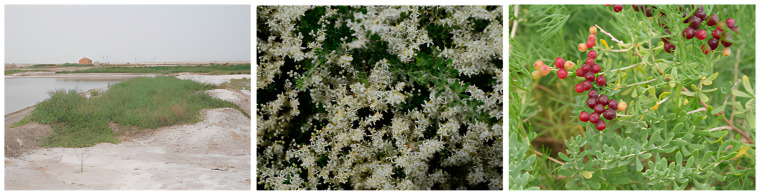
The morphological characteristics of *N. sibirica* Pall. were captured at the Yellow River Delta National Agricultural High-Tech Industrial Demonstration Zone in Binzhou, China.

Among these species, *N. sibirica* Pall. (2*n* = 2*x* = 24) stands out as an extreme xerophyte with remarkable ecological plasticity (Figure 1) [3,4,5,6,7,8,9,10]. Its distribution spans arid and semi-arid regions of Eurasia, including Mongolia, Russia (Altai Republic, Transbaikal Region), Kazakhstan, and China [1,10,11,12,13,14,15,16]. In China, it predominantly occurs in northwestern regions (Qinghai Province [5,17], Xinjiang Uygur Autonomous Region [18], and Gansu Province [16]), North China, and eastern coastal areas. Morphologically, *N. sibirica* displays distinctive adaptations such as reduced leaf size, black fruits, and exceptional floral productivity (up to 90 flowers per inflorescence) in arid environments like the III Depression [19]. Notably, it is the only *Nitraria* species inhabiting northern China’s coastal regions, where it colonizes extreme habitats including lake basin margins, salt-encrusted sandy lands, and coastal saline zones [20]. As a halophytic pioneer plant, it exhibits remarkable adaptive traits, including drought resistance, wind erosion tolerance, rapid growth, and a robust root system, which contribute to its ecological role in mitigating soil salinization and desertification [21]. Compared to other pioneer species like *Tamarix chinensis*, *N. sibirica* demonstrates superior Na^+^ accumulation capacity (6.339 vs. 4.071 mg/g DW under 300 mmol/L NaCl) and a higher Na^+^/Cl⁻ ratio (5.42 vs. 5.65), indicating enhanced salt dilution efficiency. This adaptive trait, coupled with its betaine content increasing by 18.4% under high salinity (vs. 17.6% in *T. chinensis*), enables it to stabilize hypersaline soils while maintaining metabolic function, making it a keystone species for rehabilitating degraded arid ecosystems [22]. Its exceptional stress tolerance mechanisms have established *N. sibirica* as a model organism for studying plant adaptation to extreme environments.

Beyond its ecological resilience, *N. sibirica* has attracted increasing interest in pharmaceutical and functional food research due to its rich phytochemical diversity and multifaceted bioactivities. Recent studies have revealed a complex array of bioactive compounds, including phenolic glycosides (e.g., clovin, rutin derivatives), alkaloids (e.g., nitraramine, sibirine), polysaccharides, and anthocyanins, which underpin its antioxidant, antimicrobial, and antihypertensive properties [23,24,25,26]. Comparative studies across the *Nitraria* genus highlight its unique biochemical profile, with interspecific analyses ranking *N. sibirica* among the most promising sources of bioactive compounds. For instance, while foliar flavonoid accumulation in *N. sibirica* is surpassed only by *N. sphaerocarpa* within the genus, its leaves exhibit a threefold higher flavonoid content compared to its own fruits. This preferential foliar accumulation underscores its potential as a viable source of bioactive flavonoids for therapeutic applications [27]. Furthermore, *N. sibirica* distinguishes itself through unique lipid compositions, including high levels of acetic acid—absent in closely related species like *N. tangutorum*—and elevated n-hexadecanoic acid concentrations, underscoring its phytochemical distinctiveness [28]. Traditionally, its fruit, revered as the “desert cherry” in Northwest China, has been used in Uygur and Mongolian ethnomedicine to treat hypertension, gastroenteritis, and inflammation, as documented in *Xinjiang Ethnomedicine Records* [29]. For instance, decoctions of its fruit are prescribed to regulate blood pressure, while leaf extracts are applied topically for anti-inflammatory purposes [7].

Despite growing scientific validation of its medicinal potential, a systematic synthesis of its chemical constituents, pharmacological mechanisms, and translational applications remains lacking. This review aims to consolidate current knowledge on *N. sibirica*’s phytochemistry, bioactivity, and functional food prospects, while addressing critical gaps in mechanistic understanding and clinical validation. By integrating ecological, phytochemical, and pharmacological perspectives, we seek to provide a foundation for future research and sustainable utilization of this species in nutraceutical and therapeutic development. The relevant literature was retrieved through comprehensive searches on PubMed, Web of Science, and CNKI, prioritizing studies from 2015 to 2024 to ensure up-to-date coverage.

## 2. Environmental Role of *N. sibirica*

As a pioneer species in saline–alkaline and desert ecosystems, *N. sibirica* plays a crucial role in maintaining ecological stability through multiple mechanisms. It is widely cultivated for stabilizing sand deposits and desalinizing saline soil [30]. Comparative studies with *N. tangutorum* and *N. roborowskii* reveal that *N. sibirica* exhibits superior salt tolerance, evidenced by higher biomass (12.42 g vs. 10.19–10.49 g under 400 mmol·L^−1^ NaCl), elevated chlorophyll content (0.78 mg·g^−1^), and enhanced SOD activity (84.46 U·g^−1^), which collectively enhance its resilience in extreme environments [31]. The plant’s extensive root system significantly improves soil structure, mitigates erosion, and enhances water retention in arid environments [18]. Transcriptomic studies reveal salt-induced upregulation of ethylene-responsive transcription factors (ERFs) that regulate carbon fixation genes. These findings provide genetic targets for engineering salt-tolerant crops or forestry species to combat desertification [30]. Additionally, it serves as an effective natural barrier against desertification while providing essential habitats for diverse microorganisms and fauna. Remarkably, N. sibirica demonstrates exceptional phytoremediation potential, particularly in heavy metal-contaminated soils, through its symbiotic relationship with arbuscular mycorrhizal (AM) fungi. Research has shown that association with *Funneliformis mosseae* not only increases biomass production and nutrient acquisition but also reduces sodium (Na^+^) and cadmium (Cd) accumulation in shoot tissues, making it particularly valuable for rehabilitating co-contaminated ecosystem [32]. Recent breakthroughs in multi-omics approaches, combining transcriptomic and metabolomic analyses, have further revealed the sophisticated molecular networks responsible for *N. sibirica*’s remarkable stress tolerance [33,34,35]. These findings provide critical insights into the genetic and biochemical basis of its ecological adaptability.

### 2.1. Ion Homeostasis and Transport Regulation

The species’ ability to thrive under salt–alkali stress is underpinned by a sophisticated network of physiological, molecular, and metabolic adaptations. Central to its salt tolerance is the precise regulation of ion homeostasis, achieved through the coordinated activity of specialized transporters and channels [36,37]. Complementing these transporters, vacuolar Na^+^/H^+^ exchangers (*NsNHX1*, *NsNHX4*, *NsNHX7*) sequester excess Na^+^ into vacuoles, minimizing its harmful effects in the cytosol [38]. The high-affinity potassium transporter *NsHKT1* ensures selective K^+^ uptake in roots while restricting Na^+^ translocation to aerial tissues, thereby maintaining a favorable Na^+^/K^+^ balance critical for cellular functions [39]. Similarly, the plasma membrane Na^+^/H^+^ antiporter *NsSOS1* facilitates the extrusion of cytotoxic Na^+^ from cells, a mechanism that has been successfully transferred to transgenic poplar, enhancing its salt tolerance [40]. This compartmentalization is further supported by the upregulation of vacuolar H^+^-ATPase (*NsVHA*) and H^+^-PPase (*NsVP1*), which generate the proton gradients necessary for Na^+^ storage. Concurrently, Two Pore K^+^ (TPK) channels mediate K^+^ release from vacuoles into the cytosol, ensuring K^+^ availability for essential metabolic processes. These ion transport mechanisms are often tissue-specific, with roots retaining K^+^ and leaves acting as Na^+^ sinks, a strategy that collectively safeguards the plant’s physiological integrity under salinity. Salt treatment upregulates genes (e.g., rbcL, maeB) in the “Carbon fixation in photosynthetic organisms” pathway, enhancing photosynthetic efficiency and starch accumulation. This suggests improved carbon sequestration capacity under saline conditions, further supporting its role in ecosystem rehabilitation [30].

### 2.2. Antioxidant Defense and Osmoprotectant Accumulation

To counteract the oxidative stress induced by salt and drought, *N. sibirica* activates a robust antioxidant defense system. Enzymes such as superoxide dismutase (SOD), catalase (CAT), and ascorbate peroxidase (APX) scavenge ROS, while non-enzymatic antioxidants like ascorbate (AsA) and glutathione (GSH) are elevated through the ascorbate–glutathione cycle. Exogenous application of calcium (CaCl_2_) further enhances this cycle by improving AsA/DHA and GSH/GSSG ratios, thereby boosting the plant’s ROS-scavenging capacity [41]. Osmotic adjustment is another critical survival strategy, with *N. sibirica* accumulating compatible solutes such as proline, soluble sugars, and starch to maintain cellular turgor and protect macromolecules under water deficit [42]. Metabolomic studies reveal that salt stress triggers extensive reprogramming of metabolic pathways, particularly those related to amino acid biosynthesis (e.g., valine, leucine, isoleucine) and secondary metabolism (e.g., flavonoids, phenylpropanoids) [43]. These metabolites not only serve as osmolytes but also reinforce cell walls and neutralize ROS, highlighting the integration of metabolic and antioxidant responses in stress adaptation.

### 2.3. Hormonal and Calcium Signaling

Hormonal signaling and calcium-mediated pathways further fine-tune *N. sibirica*’s stress responses. Abscisic acid (ABA) plays a central role in drought adaptation by inducing stomatal closure and promoting flavonoid synthesis, while gibberellin (GA) suppression under stress conditions redirects resources from growth to defense [43]. Calcium sensors, including calmodulin (*NsCaM*) and calmodulin-like (*NsCML*) proteins, act as molecular switches that regulate H_2_O_2_ levels and ion transport under salinity and cold stress [44]. For instance, *NsCML2*, a homolog of *Arabidopsis AtCBL2*, is implicated in vacuolar Na^+^ sequestration and exhibits dynamic expression patterns under abiotic stresses, underscoring its importance in cross-stress resilience [45].

The ecological and physiological versatility of *N. sibirica* offers promising applications in agriculture and environmental management. Its stress-tolerance genes, such as *NsHKT1* and *NsSOS1*, are prime candidates for genetic engineering to improve salt tolerance in crops and forestry species. Meanwhile, its natural ability to thrive in heavy metal–laden soils, aided by AM fungi, positions it as a sustainable tool for phytoremediation. Future research should focus on field trials of transgenic plants expressing *N. sibirica* genes, as well as large-scale restoration projects leveraging its hardiness to rehabilitate saline and degraded ecosystems. By unraveling the intricate mechanisms behind this halophyte’s resilience, scientists can harness its potential to address global challenges like soil salinization, water scarcity, and food security, paving the way for more sustainable land-use practices in a changing climate.

## 3. Chemical Composition Study

*N. sibirica* is a phytochemical-rich plant with diverse bioactive compounds, including flavonoids (e.g., clovin, rutin derivatives), alkaloids (e.g., nitraramine, sibirine), polysaccharides (α-(1 → 4)-D-galacturonan backbone), and anthocyanins (e.g., cyanidin, petunidin). Leaf extracts are particularly rich in chlorogenic acid and rutin analogs, while aerial tissues accumulate alkaloids. The plant also contains essential oils, fatty acids (e.g., oleic acid), vitamins (notably vitamin C, up to 444.07 mg/100 g in fruit juice), and minerals (e.g., Fe, Zn), with leaves as primary trace metal reservoirs. Tissue-specific and seasonal metabolite variations suggest environmental adaptation, yet gaps remain in understanding compound synergies and ecological mechanisms.

### 3.1. Phenolic Compounds

Recent advancements in phytochemical research have significantly increased interest in phenolic extracts from the *Nitraria* genus, particularly *N. sibirica*. Among its diverse phytochemical constituents, phenolic compounds, especially flavonoids and lignans, have become a major focus due to their broad-spectrum bioactivities (Table 1). Quantitative analysis of four *Nitraria* species from Inner Mongolia was conducted using aluminum nitrate-sodium nitrite spectrophotometry. The study revealed significant interspecific and tissue-specific variations in total flavonoid content (*p* < 0.05). Among fruits, *N. sphaerocarpa* exhibited the highest concentration (1.587 ± 0.003%), followed by *N. sibirica* (1.425 ± 0.004%) and *N. roborowskii* (1.494 ± 0.001%), with *N. tangutorum* showing the lowest levels (1.376 ± 0.002%). In foliar tissues, *N. sphaerocarpa* again demonstrated superior flavonoid accumulation (1.772 ± 0.005%), while *N. sibirica* leaves contained 0.131 ± 0.005% flavonoids—approximately threefold higher than its fruit content (0.042 ± 0.002%). These findings highlight *N. sibirica* as one of the most promising flavonoid sources, with its leaves representing a secondary reservoir of these bioactive compounds. This preferential foliar accumulation suggests *N. sibirica* leaves may represent a more viable source of bioactive flavonoids than its fruits for potential therapeutic applications, despite not being the highest-yielding species in the genus. The 3:1 leaf-to-fruit flavonoid ratio provides clear guidance for targeted phytochemical exploitation of this species [27]. Habitat-dependent variations in phenolic profiles were identified using high-performance liquid chromatography (HPLC), reflecting not only environmental influences on metabolite biosynthesis but also potential genetic or epigenetic adaptations within *N. sibirica* populations [46]. Similar to other plant species, ecological conditions can modulate phytochemical composition and diversity [47,48]. These variations may impact key biological processes such as sodium ion homeostasis, osmotic stress response, ROS scavenging, cell wall stabilization, signal transduction, and photosynthetic efficiency, all of which are critical for enhancing salt tolerance [46]. Building on this, Turghun et al. isolated 16 phenolic compounds from *n*-BuOH leaf extracts, including nikoenoside, roseoside II, and vanilloloside, validated through NMR and MS spectral analysis [49]. Advanced UHPLC-Q-Orbitrap-MS further resolved the phenolic architecture of *N. sibirica* leaf extracts (NSL-EPE), revealing a tripartite composition dominated by flavonoids (e.g., clovin, rutin-7-O-α-rhamnoside), phenylpropanoids (e.g., chlorogenic acid), and lignans (e.g., syringin), which collectively constitute >60% of the phenolic fraction [23]. Notably, lignan-rich glycosides exhibited the highest bioactivity in leaves, suggesting their therapeutic primacy [50]. Flavonoids, the most structurally diverse phenolic subclass, were further enriched by Turghun et al., who isolated eight derivatives—including diosmin and luteolin—from ethyl acetate (EtOAc) leaf extracts [51,52]. These findings highlight *N. sibirica*’s potential as a source of novel flavonoids for nutraceutical development.

**Table 1:** Flavanoid compounds isolated from the *N. sibirica*.

No.	Compound	Part of Plant	Molecular Formula	Reference
1	Rutin-7-O-α-L-rhamnopyranoside	Leaves	C_33_H_40_O_20_	[51]
2	Clovin	Leaves	C_33_H_40_O_20_	[51]
3	Rutin	Leaves; fruit	C_27_H_30_O_16_	[51]
4	Narcissin	Leaves	C_28_H_32_O_16_	[51]
5	Diosmin	Leaves	C_28_H_32_O_15_	[51]
6	Quercetin-7-O-α-L-rhamnopyranoside	Leaves	C_21_H_20_O_11_	[51]
7	Diosmetin	Leaves	C_16_H_12_O_6_	[51]
8	Luteolin	Leaves	C_15_H_10_O_6_	[51]
9	3,5-dimethoxykaempferol-7-O-glucoside	Fruit	C_23_H_24_O_11_	[52]
10	Chryseriol-3-O-rutinoside	Fruit	C_28_H_32_O_15_	[52]

### 3.2. Alkaloids

*N. sibirica* is distinguished by its remarkable alkaloid diversity, with over 25 structurally unique compounds identified to date, including nitraramine, sibirine, and schoberine (Table 2). The study of *N. sibirica* alkaloids has evolved significantly since their initial isolation [24,25,53,54]. Subsequent research expanded the alkaloid catalog through column chromatography of benzene-soluble fractions [55,56]. Aerial tissues, particularly leaves, exhibit markedly higher alkaloid concentrations compared to fruits, likely due to elevated biosynthetic activity in photosynthetic organs [57]. Advanced extraction techniques, such as pH-zone-refining counter-current chromatography (pH-ZRCCC), have enabled the high-purity isolation of novel alkaloids, revealing significant spatial heterogeneity in their distribution [58]. Standardized protocols using atropine sulfate as an internal reference confirmed tissue-specific alkaloid accumulation patterns, solidifying leaves as the primary biosynthetic reservoirs [33].

Beyond alkaloid profiling, recent studies highlight dynamic seasonal fluctuations in secondary metabolites. During fruiting stages, leaves exhibit increased levels of flavonoids and tannins, alongside a sharp decline in catechin levels [57], suggesting adaptive metabolic responses to environmental stressors. These findings underscore the need to investigate how abiotic factors—such as temperature, light, and soil salinity—modulate phytochemical profiles, as these variations directly impact the plant’s medicinal efficacy and industrial applicability.

**Table 2:** Alkaloids compounds isolated from the *N. sibirica*.

No.	Compound	Part of Plant	Molecular Formula	Reference
1	Nitraramine	Aerial parts	C_15_H_24_N_2_O	[24,56]
2	Isonitramine	Aerial parts	C_10_H_19_NO	[53]
3	Sibirine	Aerial parts	C_11_H_21_NO	[25]
4	Nitrabirine	Aerial parts	C_12_H_18_N_2_O	[54,55]
5	Nitraramine N-oxide	Aerial parts	C_15_H_24_N_2_O_2_	[55]
6	Deoxyvasicinone	Aerial parts	C_11_H_10_N_2_O	[55]
7	Schoberine	Aerial parts	C_15_H_26_N_2_	[55]
8	Dehydroschoberine	Aerial parts	C_15_H_24_N_2_	[55]
9	Dihydroschoberine	Aerial parts	C_15_H_28_N_2_	[55]
10	Nitrabirine N-oxide	Aerial parts	C_12_H_18_N_2_O_2_	[55]
11	Nitraramidine	Aerial parts	C_23_H_30_N_2_O_3_	[56]
12	Nitraraidine	Aerial parts	C_20_H_25_N_2_	[56]
13	Isonitramine	Aerial parts	C_10_H_19_NO	[56]
14	L-vasicinone	Aerial parts	C_11_H_10_N_2_O_2_	[56]
15	Nitraroxine	Aerial parts	C_15_H_24_N_2_O_2_	[56]
16	Sibirinine	Aerial parts	C_12_H_21_NO_2_	[56]
17	Schobemine	Leaves	C_15_H_24_N_2_	[58]
18	Schoberidine	Leaves	C_20_H_21_N_3_	[58]
19	Schoberimine	Leaves	C_22_H_25_N_3_	[58]
20	N-malonyl-tryptophan	Fruit	C_14_H_14_N_2_O_5_	[59]
21	5-(methoxymethyl)-1H-pyrrole-2-carbaldehyde	Fruit	C_7_H_9_NO_2_	[59,60]
22	2-[2-formyl-5-(methoxymethyl)-1H-pyrrol-1-yl]-propanoate	Fruit	C_11_H_15_NO_4_	[59,61]

### 3.3. Polysaccharides

Pectic polysaccharides extracted from *N. sibirica* via ammonium oxalate yielded 2.0% (*w*/*w*) of dry biomass. Compositional analysis revealed a galacturonic acid-rich structure (62.5%), with arabinose (19.9%), rhamnose (3.1%), and galactose (3.6%) [26]. The polysaccharides exhibited high polydispersity (*M*_w_/*M*_n_ = 14.6), indicative of structural heterogeneity, and featured a backbone of α-(1 → 4)-D-galacturonan with branched L-arabinofuranose residues in terminal, (1 → 5)-, (1 → 3)-, and 3,5-substituted configurations. Physicochemical profiling identified three molecular weight fractions (1.72 million, 240,000, and 130,000 Da) and a uronic acid content of 15.84%, which enhances solubility and bioactivity. Notably, glucose emerged as the dominant monosaccharide (28.71%), underscoring its role in maintaining structural stability [62,63].

Further purification of *N. sibirica* polysaccharides (NSP) via ultrasonic-enzyme-assisted extraction (UEAE) and DEAE-52 column chromatography yielded three fractions: NSP-1, NSP-2, and NSP-3 [63]. Comprehensive characterization, including chemical composition, molecular weight profiling, monosaccharide content, glycosidic linkage analysis, and assessments of surface morphology and thermal stability, revealed their structural architecture. The fractions were identified as homogeneous heteropolysaccharides with distinct molar ratios of rhamnose (Rha), arabinose (Ara), mannose (Man), glucose (Glc), and galactose (Gal). Neutral fraction NSP-1 was dominated by Man, followed by Glc and Gal, while acidic fractions NSP-2 and NSP-3 primarily contained Rha, with Gal as the secondary component [63]. These structural distinctions highlight the influence of extraction and purification methods on polysaccharide functionality, providing insights for tailored applications in nutraceuticals or biomaterials.

### 3.4. Pigments

*N. sibirica* is a rich source of natural pigments, including anthocyanins, proanthocyanidins, and red flavonoid-derived compounds (e.g., arachidoside) [64,65,66,67,68]. These pigments hold significant potential as stable, eco-friendly alternatives to synthetic colorants in acidic beverages and functional foods.

Red pigments demonstrate dual solubility in water and ethanol, with optimal stability under mildly acidic conditions (pH ≤ 4.62). Extraction using 0.3% hydrochloric acid yields superior results, making it suitable for acidic food and beverage applications. Stability is further enhanced under low light and refrigeration, with protection from oxidative agents (e.g., hydrogen peroxide) and metal ions recommended to prevent degradation [64,65,66]. Moreover, structural characterization identified 12 anthocyanin derivatives, including cyanidin 3-O-sophoroside, petunidin 3-O-rhamnoside, and malvidin 3-O-arabinose [67]. Subcritical water extraction (SWE) optimized anthocyanin isolation, revealing eight distinct derivatives of cyanidin, petunidin, delphinidin, and pelargonidin [68]. Transcriptomic analyses have identified R2R3-MYB transcription factors (NsMYB1 and NsMYB5) as key regulators of anthocyanin biosynthesis [69,70]. Cyanidin-3-[2″-(6‴-coumaroyl)-glucosyl]-glucoside was identified as the predominant anthocyanin in black fruits, with NsMYB1 regulating its biosynthesis [69]. NsMYB5 as a positive regulator of anthocyanin and proanthocyanidin synthesis, elucidating the molecular basis for color differentiation between red and black fruits [70]. These findings position *N. sibirica* as both a sustainable pigment source and a model for studying plant color evolution under environmental stress.

### 3.5. Lipids and Volatile Oils

Research on *N. sibirica* esters has increasingly focused on ethyl acetate and lipids due to their industrial and nutritional significance. Advanced extraction techniques, such as supercritical CO_2_ extraction combined with solvent immersion, have been successfully employed to isolate fruit oil, revealing a rich profile of bioactive lipids [71]. GC-MS analysis identified 26 compounds in the fruit oil, including 9,12-octadecadienoic acid, oleic acid, and γ-vitamin E, underscoring its potential as a functional oil source [71].

Specifically, the total fatty acid content in *N. sibirica* fruit oil was found to be 70.01 mg/g (dry weight). Among these, unsaturated fatty acids are predominant, with linoleic acid (C18:2) at 36.34 mg/g, oleic acid (C18:1) at 23.57 mg/g, and linolenic acid (C18:3) at 2.01 mg/g. The main saturated fatty acid is palmitic acid (C16:0), present at 7.65 mg/g. The ratio of unsaturated to saturated fatty acids is approximately 4.8:1, highlighting the nutritional value of N. sibirica oil compared to many conventional seed oils [72].

Leaf volatile oils, characterized by solid-phase microextraction (SPME) and GC-MS, contain over 30 compounds, with 5,6,7,7a-tetrahydro-4,4,7a-trimethyl-2(4H)-benzofuranone as the dominant aromatic compound. Notably, N. sibirica contained 20.52% acetic acid (absent in N. tanguiorum) and higher levels of n-hexadecanoic acid than that in N. tanguiorum(6.34% vs. 1.11%), while hydrocarbons were less abundant (12.00% vs. 25.54%) [28].

Unsaturated fatty acids (oleic, linoleic, and linolenic acids) dominate in leaves, exceeding levels in sarcocarps and seeds, while palmitic acid is the primary saturated fatty acid [72]. In leaves, unsaturated fatty acids account for more than 80% of the total fatty acids, with oleic and linoleic acids being the most abundant [72]. An efficient HPLC/QTOF-MS/MS method was developed to analyze the ethyl acetate fraction of *N. sibirica* fruits, identifying 28 distinct components, including 7 cinnamic acid derivatives (e.g., ferulic acid esters), 9 benzoic acid derivatives (e.g., vanillic acid glycosides), and 12 flavonoids (e.g., quercetin-3-O-glucoside) [73]. This method enhances precision in profiling *N. sibirica*’s lipidome, supporting its application in nutraceutical and food additive development.

### 3.6. Protein and Amino Acids

*N. sibirica* is also a rich source of crude protein and amino acids. Systematic nutritional evaluation revealed a consistent distribution pattern, with leaves exhibiting the highest crude protein content (leaf > fruit > branch). The plant contains 18 amino acids, including all 8 essential amino acids required for human nutrition [74]. Complementary studies confirmed high levels of free amino acids, further underscoring its nutritional value [75]. Comprehensive profiling of fruit amino acids demonstrated that essential amino acids constitute 26.62% of the total protein content, meeting FAO/WHO standards and indicating high protein quality [76]. A comparative study of leaves and branches identified aspartic acid, glutamic acid, alanine, and proline as the predominant amino acids. Except for proline, amino acid concentrations were significantly higher in leaves than in branches. Consistent with earlier findings, leucine and the methionine + cystine pair were identified as the first limiting EAAs, suggesting targeted supplementation could optimize its nutritional profile [74,77]. These findings position *N. sibirica* as a promising plant-based protein source for addressing global malnutrition and functional food development.

### 3.7. Other Constituents

The fruits and leaves of *Nitraria sibirica* are rich in vitamins, minerals, and other bioactive components, underscoring their multifunctional applications. Spectrophotometric analysis using 2,6-dichloroindophenol revealed substantial vitamin C content in fruits (46.0 mg/100 g), positioning them as excellent raw materials for food processing, especially in the production of raw juice and fruit paste [76,78]. Remarkably, fruit juice contains exceptionally high vitamin C levels (up to 444.07 mg/100 g), making it a potent resource for nutrient-dense beverages [20]. Moreover, the elemental composition of *N. sibirica* has been systematically studied, revealing distinct distribution patterns across its tissues. Leaves are primary mineral reservoirs, with elevated levels of Mn, Cr, and Cd, while Zn accumulates preferentially in branches and fruit cores [78,79]. Principal component analysis (PCA) highlighted Mn as the dominant mineral in leaves, followed by Cu, Zn, and Fe, reflecting its ecological adaptation to nutrient-poor soils [79]. Fruits exhibit substantial mineral content, with combined Fe, Ca, and Zn concentrations reaching 755.91 mg/100 g, meeting dietary mineral requirements [78]. Beyond human nutrition, *N. sibirica*’s tender stems and leaves are valuable for animal feed, containing 28.80% crude protein, 8.86% fiber, and 2.24% fat. Additionally, its seeds, rich in health-promoting lipids, offer potential for producing functional cooking oils with enhanced nutritional profiles [20]. These findings underscore *N. sibirica*’s versatility as a sustainable resource for food, feed, and industrial applications.

## 4. Pharmacological Effects

**Figure 2:**
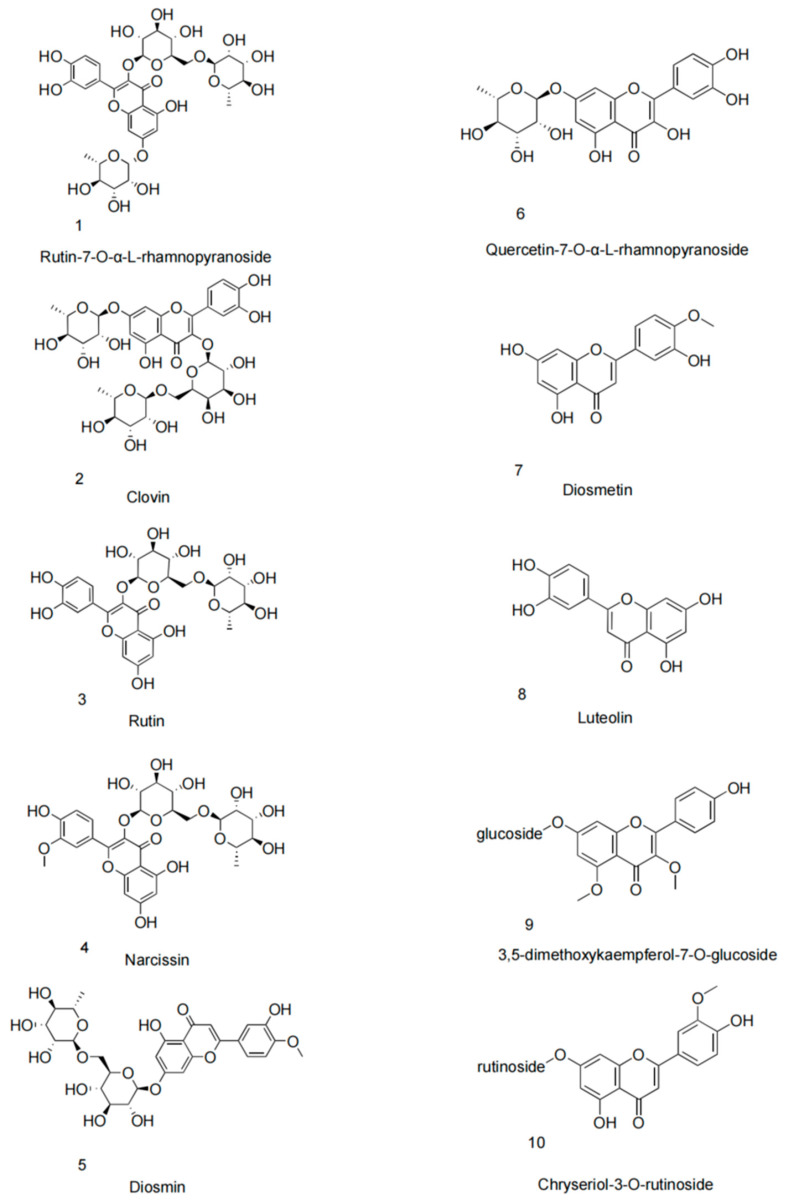
Structure of flavanoid compounds isolated from the *N. sibirica*.

**Figure 3:**
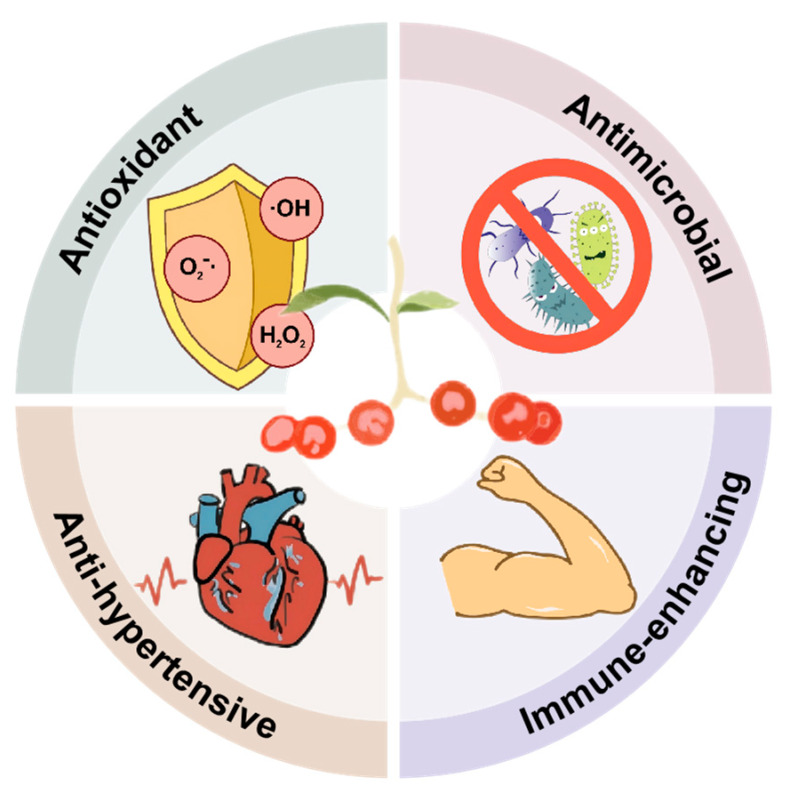
Healthy benefits of *N. sibirica*.

*N. sibirica* has emerged as a focal point in pharmacological research due to its multifaceted bioactivities, including antioxidant, antimicrobial, antihypertensive, and immunomodulatory effects (Figure 2). These activities are attributed to its rich repertoire of bioactive constituents including phenolic compounds, alkaloids, polysaccharides, and trace elements. This section synthesizes advancements in pharmacological mechanisms, active constituents, and translational applications, emphasizing compound synergies (e.g., flavonoid-alkaloid interactions) and ecological adaptability (e.g., drought-induced metabolite upregulation). By bridging ethnopharmacology with modern omics technologies, *N. sibirica* is poised to revolutionize nutraceutical and functional food industries. The diverse healthy benefits of *N. sibirica* are further illustrated in Figure 3. A summary of the pharmacological activities reported for *N. sibirica* in multiple previous studies is provided in Table 3.

### 4.1. Antioxidant Activity

Reactive oxygen species (ROS), including superoxide anions (O_2_^−^·), hydroxyl radicals (·OH), and hydrogen peroxide (H_2_O_2_), are natural byproducts of cellular metabolism. These molecules trigger lipid peroxidation, damaging cellular membranes and contributing to age-related diseases, cancer, and cardiovascular disorders [80]. Studies on *N. sibirica* highlight its potent antioxidant capacity, primarily due to polyphenol- and flavonoid-rich fractions. Aqueous-phase extracts, particularly those containing isorhamnetin-3-O-β-L-rutinoside and isorhamnetin-3-O-rutinoside-7-O-glucoside, showed superior radical-scavenging efficacy in DPPH and ABTS assays compared to other solvent extracts [81]. Freeze-dried polysaccharides also demonstrated significant scavenging activity against O_2_^−^· and DPPH radicals, outperforming hot-dried counterparts, emphasizing the importance of extraction and processing methods [82]. The crude polysaccharides extracted from *N. sibirica*, *Lycium barbarum* L., and *L. ruthenicum* Murr. in Qinghai Province were comparatively studied for their bioactivities. *N. sibirica* polysaccharides demonstrated superior DPPH radical scavenging capacity (IC_50_: 0.19 mg/mL) compared to *L. barbarum* (0.99 mg/mL) and *L. ruthenicum* (1.22 mg/mL), likely attributable to their distinct monosaccharide composition featuring higher glucose (28.71%) and galacturonic acid (11.94%) contents. All three polysaccharides exhibited immunoenhancing effects by significantly promoting Nitric Oxide (NO) secretion in macrophages. However, while Lycium polysaccharides showed pro-inflammatory potential by augmenting LPS-induced NO production, *N. sibirica* polysaccharides did not exhibit this effect, suggesting a safer immunomodulatory profile. The observed bioactivities correlate with structural characteristics such as molecular weight (130,000–2,350,000 Da) and monosaccharide ratios, as confirmed by HPLC analysis. These findings highlight *N. sibirica* polysaccharides as promising natural antioxidants with balanced immunomodulatory properties, warranting further investigation into their structure–activity relationships and potential applications in functional foods or therapeutics [62]. Additionally, subcritical water extraction (SWE) isolated anthocyanins with exceptional antioxidant properties, surpassing ascorbic acid in DPPH scavenging capacity and showing potent α-glucosidase inhibitory effects, 14-fold more effective than acarbose [68]. Molecular docking and dynamics simulations confirmed stable interactions between these anthocyanins and α-glucosidase, mediated by van der Waals forces and hydrogen bonds, highlighting their potential as natural antioxidants and anti-diabetic agents [68].

Quantitative analysis using UPLC-QQQ/MS identified tryptophan and alcesefoliside as key contributors to antioxidant activity, providing a basis for quality assessment of *N. sibirica* fruit [52]. Optimization of ultrasonic-assisted extraction through response surface methodology maximized total flavonoid content (TFC), antioxidant capacity (DPPH), and anti-proliferative effects against 3T3-L1 preadipocytes in leaf extracts [83]. These findings underscore the therapeutic potential of *N. sibirica* as a source of natural antioxidants and nutraceuticals.

### 4.2. Antimicrobial Activity

*N. sibirica* exhibits significant antibacterial properties against foodborne pathogens, primarily attributed to its polyphenol-rich fractions. Ethyl acetate extracts from fruits demonstrate potent activity against *Escherichia coli* (*E. coli*), *Bacillus subtilis* (*B. subtilis*), and *Staphylococcus aureus* (*S. aureus*), with inhibition zones of 12–18 mm in disk diffusion assays [81,84]. Systematic fractionation of N. sibirica fruit extracts revealed differential antimicrobial potency across solvent partitions. Following sequential extraction with 95% ethanol, the crude extract was partitioned using solvents of increasing polarity (petroleum ether, chloroform, ethyl acetate, and n-butanol). Disk diffusion assays demonstrated superior antibacterial activity in the ethyl acetate fraction, which exhibited minimum inhibitory concentrations (MICs) of 25–50 mg/mL against target pathogens. This finding aligns with the enrichment of bioactive polyphenols in the intermediate-polarity fraction, substantiating their crucial role in antimicrobial efficacy [84]. The consistent absence of antifungal activity across studies suggests selective targeting of bacterial systems, likely due to structural specificity of polyphenolic constituents [81]. Hydroxyl groups and conjugated π-systems in phenolic compounds (particularly cinnamic acid derivatives) facilitate membrane interaction, increasing permeability and promoting ROS accumulation. Simultaneously, flavonoid components exhibit DNA gyrase inhibition, effectively blocking bacterial nucleic acid synthesis. Comprehensive studies reveal three primary antibacterial pathways: (1) structural disruption of bacterial membranes through interactions with lipophilic domains and porin proteins, resulting in efflux of critical intracellular components (K^+^, ATP); (2) induction of oxidative stress via ROS-mediated damage to essential macromolecules; and (3) interference with quorum-sensing systems (notably acylhomoserine lactone signaling), leading to suppressed biofilm formation and attenuated virulence [85]. This targeted activity, combined with the plant’s natural origin, positions *N. sibirica* extracts as promising clean-label alternatives to synthetic preservatives, addressing growing consumer demand for sustainable food additives while maintaining safety standards.

### 4.3. Anti-Hypertensive Activity

Hypertension affects over 1.15 billion adults worldwide, driving increased interest in natural therapeutics like *N. sibirica* Pall., a traditional medicinal plant used in Xinjiang, China for blood pressure regulation [86,87]. Modern research has elucidated its multi-target mechanisms of action including antioxidant stress, angiotensin-converting enzyme (ACE) inhibition, endothelium-dependent vasorelaxation via nitric oxide synthase (NOS) activation, and mitigation of hypertension-induced renal damage. For example, Leaf extracts of *N. sibirica* lowered blood pressure in SHR models, likely by countering oxidative stress-induced endothelial dysfunction, and improved lipid profiles (reduced plasma total cholesterol, triglycerides, urea nitrogen, and creatinine) [23]. Aqueous extracts demonstrated significant ACE inhibitory activity (IC_50_ = 55.85 g/L), validated through HPLC analysis of hippuric acid levels [88]. Moreover, hydroalcoholic fruit extracts of *N. sibirica* induced vasodilation via NOS activation, endothelial-derived hyperpolarizing factor (EDHF) release, and muscarinic receptor stimulation. In vivo studies confirmed blood pressure reduction in spontaneously hypertensive rats (SHR) and Wistar-Kyoto (WKY) rats [89]. Total alkaloids from leaves reduce albuminuria (albumin in urine, a marker of kidney damage) in mice fed a high-salt diet with angiotensin II. This effect is linked to attenuated renal inflammation and fibrosis biomarkers, though specific bioactive alkaloids remain unidentified [90]. These findings position *N. sibirica* as a promising multi-target therapeutic candidate for hypertension management, particularly given its additional benefits on lipid metabolism and renal protection. However, critical gaps remain in the development of *N. sibirica*-derived ACE inhibitors as clinically viable antihypertensive agents. Notably, future research priorities should include comprehensive pharmacokinetic profiling of bioactive constituents and systematic safety evaluation.

### 4.4. Other Activities

Beyond its primary pharmacological effects, *N. sibirica* exhibits diverse bioactivities that underscore its therapeutic versatility. Crude polysaccharides significantly enhance NO secretion in murine peritoneal macrophages (*p* < 0.05), indicating immunomodulatory potential [62]. The plant’s pectin components demonstrate anti-allergenic properties by inhibiting ovalbumin (OVA) absorption in mice through non-covalent interactions (electrostatic and hydrophobic forces), reducing serum OVA levels by 40–50% (*p* < 0.01) [26]. Additionally, flavonoid-rich extracts show anti-obesity potential by suppressing adipogenesis in 3T3-L1 cells by 40% (*p* < 0.05), likely through PPARγ downregulation and AMPK activation [83]. These findings—spanning immunomodulation, anti-allergy, and metabolic regulation—highlight *N. sibirica*’s promise as a source of functional food ingredients and therapeutic agents for metabolic and immune-related disorders. Further research should focus on compound isolation, clinical validation, and mechanistic studies to fully exploit its multifunctional applications.

**Table 3:** Summary of *N. sibirica* parmacological activities reported in multiple previous studies.

Pharmacological Activity	Key Findings/Data	Reference
Antioxidant	DPPH radical scavenging (IC_50_: 0.19 mg/mL; superior to *L. barbarum* and *L. ruthenicum*); Significant O_2_^−^· and DPPH scavenging by freeze-dried polysaccharides; Anthocyanins from subcritical water extraction stronger than ascorbic acid and 14x acarbose	[62,68,81,82]
Antimicrobial	Ethyl acetate extract: inhibition zones 12–18 mm (*E. coli*, *B. subtilis*, *S. aureus*); MIC: 25–50 mg/mL; No antifungal activity	[81,84]
Anti-hypertensive	Leaf extracts reduce BP in SHR models; Aqueous extract ACE inhibitory (IC_50_ = 55.85 g/L); Fruit extracts induce vasodilation via NOS; Total alkaloids reduce albuminuria in mice fed high-salt diet with Ang II	[23,88,89,90]
Immunomodulatory	Polysaccharides promote NO secretion in macrophages, immune enhancement; No pro-inflammatory effect unlike *Lycium* polysaccharides	[62]
Anti-obesity	Flavonoid-rich extract inhibits adipogenesis by 40% in 3T3-L1 cells	[83]
Hypoallergic effect	Pectin from *N. sibirica* inhibits ovalbumin absorption in mice (reduced serum OVA)	[26]

## 5. Conclusions

**Figure 4:**
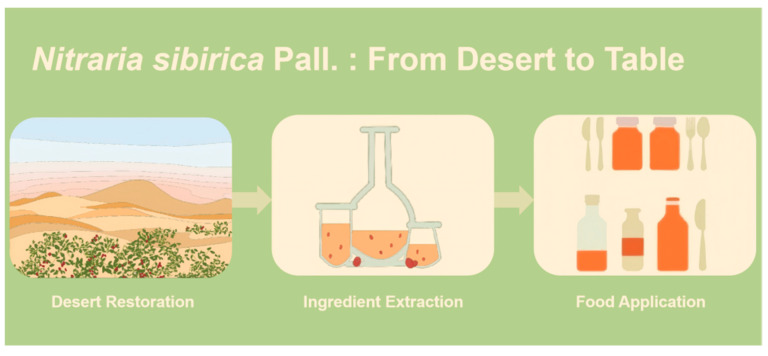
*Nitraria sibirica* Pall.: From desert to table.

*N. sibirica* Pall. emerges as a promising multifunctional plant resource with significant potential for food and nutraceutical applications. The plant’s rich phytochemical profile, including flavonoids, alkaloids, polysaccharides, and anthocyanins, underpins its diverse bioactivities. Particularly noteworthy is its exceptional antioxidant capacity, with polysaccharides demonstrating superior DPPH radical scavenging activity (IC_50_: 0.19 mg/mL) compared to related species. The plant’s antimicrobial properties, evidenced by inhibition zones of 12–18 mm against foodborne pathogens, position it as a potential natural preservative. Furthermore, its antihypertensive effects, mediated through ACE inhibition and vasorelaxation mechanisms, highlight its therapeutic potential. The high nutritional value of its fruits, containing essential amino acids (26.62% of total protein) and vitamin C (up to 444.07 mg/100 g in fruit juice), underscores its suitability for functional food development. To better illustrate the multifaceted value of *N. sibirica*, a comprehensive value chain diagram is presented (Figure 4), depicting the journey of this plant from its role in ecological restoration in desert environments, through the extraction and utilization of its bioactive components, to its final applications in the development of functional foods and nutraceutical products.

Future research should focus on optimizing extraction methods, elucidating compound synergies, and developing standardized formulations for food and nutraceutical applications. The integration of traditional knowledge with modern scientific approaches will be crucial for harnessing the full potential of this valuable plant resource in the food industry. Despite these advancements, the lack of clinical trial data remains a major limitation. Future research should focus on addressing this gap; for example, through safety assessment, pharmacokinetic studies, and biomarker-based efficacy evaluation in human subjects. Furthermore, *N. sibirica*’s ecological traits (e.g., sand fixation) and stress-resistance genes (e.g., NsMYB1, NsHKT1) offer great potential for breeding stress-tolerant crops and engineering phytoremediation systems. However, the use of these genes in transgenic systems also raises important biosafety concerns, including the possibility of gene flow, ecological impacts, and the need to comply with regulatory frameworks. Addressing these considerations is essential for the responsible development and application of *N. sibirica* resources. Interdisciplinary collaboration across ecology, pharmacology, and agronomy will further facilitate the integration of *N. sibirica* into traditional medicine, functional food innovation, and ecological restoration, thus contributing to sustainable solutions for global health and environmental challenges.

## Data Availability

No new data were created or analyzed in this study. Data sharing is not applicable to this article.
